# The Recycling Endosome in Nerve Cell Development: One Rab to Rule Them All?

**DOI:** 10.3389/fcell.2020.603794

**Published:** 2020-12-10

**Authors:** Victoria Rozés-Salvador, Christian González-Billault, Cecilia Conde

**Affiliations:** ^1^Instituto de Investigación Médica Mercedes y Martín Ferreyra INIMEC-CONICET-UNC, Córdoba, Argentina; ^2^Instituto de Ciencias Básicas, Universidad Nacional de Villa María, Córdoba, Argentina; ^3^Department of Biology, Faculty of Sciences, Universidad de Chile, Santiago, Chile; ^4^Department of Neurosciences, Faculty of Medicine, Universidad de Chile, Santiago, Chile; ^5^Geroscience Center for Brain Health and Metabolism, Santiago, Chile; ^6^The Buck Institute for Research on Aging, Novato, CA, United States

**Keywords:** Rabs, neuronal development, recycling endosome, endosomal pathway, development, Arf6, Rab35, Rab11

## Abstract

Endocytic recycling is an intracellular process that returns internalized molecules back to the plasma membrane and plays crucial roles not only in the reuse of receptor molecules but also in the remodeling of the different components of this membrane. This process is required for a diversity of cellular events, including neuronal morphology acquisition and functional regulation, among others. The recycling endosome (RE) is a key vesicular component involved in endocytic recycling. Recycling back to the cell surface may occur with the participation of several different Rab proteins, which are master regulators of membrane/protein trafficking in nerve cells. The RE consists of a network of interconnected and functionally distinct tubular subdomains that originate from sorting endosomes and transport their cargoes along microtubule tracks, by fast or slow recycling pathways. Different populations of REs, particularly those formed by Rab11, Rab35, and Arf6, are associated with a myriad of signaling proteins. In this review, we discuss the cumulative evidence suggesting the existence of heterogeneous domains of REs, controlling different aspects of neurogenesis, with a particular focus on the commonalities and singularities of these REs and their contribution to nerve development and differentiation in several animal models.

## Introduction

During neurodevelopment, dynamic morphological changes occur in migration, neurite outgrowth, dendritic spine formation, axon myelination, and synaptogenesis. These processes involve synthesis and classification of specific proteins, redistribution of cellular components, and membrane addition, among others. For this, the correct coordination and synchronization of the endosomal traffic machinery is essential (Platta and Stenmark, 2011).

Recycling endosomes (REs) play an important role in the reuse of receptor molecules as well as in the remodeling of the protein and lipid composition of the plasma membrane. Specifically, in neurons, they regulate retrograde neurotrophic signaling, axonal pathway fixation during protein development, renewal and degradation, vesicle recycling, and synaptic plasticity, among other processes (Kennedy and Ehlers, 2006; Dittman and Ryan, 2009; Winckler and Mellman, 2010; Itofusa and Kamiguchi, 2011). The morphology, distribution, and function of REs in polarized cells are different compared with other cells, especially with regard to their sorting ability, and in their recruitment of Rab proteins and adapters (Thompson et al., 2007; Fields et al., 2010). Due to the spatial demands of the neuron, REs are spread throughout soma, dendrites, and axons, unlike in non-neuronal cells where they are clustered tightly near the nucleus (Prekeris et al., 1999; Park et al., 2006; Ascano et al., 2009).

One of the best-characterized families related to the endosomal pathway is the Ras superfamily of small guanosine triphosphatase (small GTPases) related proteins, functioning as GDP/GTP-regulated molecular switches (Vetter and Wittinghofer, 2001). Based on sequence and similarity, Ras can be divided into five major classes: Ras, Rho, Rab, Ran, and Arf small GTPases.

Both Rab and Arf proteins, in their active state (bound to GTP), recruit endosome membrane-specific effector proteins (Novick and Zerial, 1997; Panopoulou et al., 2002) and are found in different subsets of membrane domains along the secretory and endocytic pathways. Recent roles for endosomal recycling pathways have been identified: (a) in the exocytic transport where exocytic proteins traverse through REs before their delivery to the plasma membrane (PM) (Ang et al., 2004; Cresawn et al., 2007; Misaki et al., 2010); (b) in retrograde transport, where cargoes internalized from PM, must pass through REs to reach the Golgi (Uchida et al., 2011; Bai and Grant, 2015); and (c) in degradation transport where REs participate to degradation or promoting autophagy (Husebye et al., 2010; Matsui et al., 2011; Longatti et al., 2012). However, these RE’s roles have not been described in neuronal cells.

Different markers have been associated with REs, such as Rab11, Rab35, and Arf6 (Calhoun and Goldenring, 1996). Some of the regulatory functions described for Rabs include the interaction with effector proteins that select cargo, the promotion of movement of vesicles to different compartments, and the verification of the correct fusion site. In addition, Rabs interact with GEFs (nucleotide exchange factors) or GAPs (GTPase-activating proteins) that act as activators or negative regulators, respectively (Hutagalung and Novick, 2011).

In this review, we analyze the accumulated evidence regarding different Rabs that share heterogeneous and dynamics domains in the RE, with an emphasis on Rab11, Rab35, and Arf6 and how they control the different cellular functions associated with neuronal development and differentiation in several models.

## Brief Description of Rab35, Rab11, and Arf6 Expression

Rab35 transcripts are expressed ubiquitously and at similar levels in all major human tissues. The gene is evolutionarily conserved, with homologs present in invertebrates and other lower organisms. This suggests that Rab35 has important and general cellular and/or developmental functions (Zhu et al., 1994).

Arfs (ADP ribosylation factors) are expressed in all eukaryotes. There are six mammalian Arfs and many more Arf-like proteins. Arf6 has distinct peripheral membrane distributions and diverse cellular activities. Mammalian Arf6 homologs exist in almost all eukaryotes (Cavenagh et al., 1996; Al-Awar et al., 2000).

Rab11 is a GTPase encoded by three different genes, *Rab11a*, *Rab11b*, and *Rab25*, whose proteins are ubiquitously expressed; enriched in the brain, heart, and testis; or restricted to epithelia, respectively (Bhartur et al., 2000). In recent years, Rab11 has emerged as an important modulator of cellular transport by regulating the association of REs with trafficking vesicles, allowing the delivery of cellular components or signaling molecules to specific locations in the cell (Jing and Prekeris, 2009).

## Rab11, Rab35, and Arf6 Re-Associated Functions During Mammalian Neurodevelopment

Neural development refers to those changes that occur in a cell from a completely undifferentiated stage to a differentiated or mature stage. Thus, we have focused on five aspects that we consider relevant to neuronal development, such as axonal and neurite growth, dendritic growth, migration of cortical neurons, synaptic plasticity, and glial differentiation and myelination.

### Neurite Outgrowth and Axon Elongation

Neurite outgrowth is a process by which developing neurons produce new projections as they grow. In this regard, Rab35 favors axon elongation in rat primary neurons in an activity-dependent manner. In this regard, p53-related protein kinase (PRPK) negatively regulates axonal elongation by reducing Rab35 protein levels through the ubiquitin-proteasome degradation pathway (Villarroel-Campos et al., 2016b).

Studies in immortalized neuronal cells show that Rab35-induced neurite growth and baseline levels of neurite extension are attenuated by loss of function of Rab35 (using dominant-negative Rab35S22N or siRNA Rab35) (Chevallier et al., 2009). From these early studies, it became evident that Rab35 is key for coordinating and recruiting downstream Rab GTPases. A reciprocal control between Rab35 and Arf6 GTPases which is an important switch to control receptor recycling during cell migration (Allaire et al., 2013) and successful cytokinesis (Chesneau et al., 2012) has been proposed. Such regulatory mechanisms would be also essential to propelling neurite elongation. In this regard, ACAP2 (also known as centaurin-β2) functions as a Rab35 effector and as an Arf6-GAP during neurite growth. Rab35 accumulates in Arf6-positive endosomes in response to stimulation of the nerve growth factor (NGF), and ACAP2 is recruited into the same compartment in a Rab35-dependent manner (Kobayashi and Fukuda, 2012). By using siRNA, it was demonstrated that both Rab35 and MICAL-like protein 1 are necessary for the localization of Rabs 8, 13, and 36 in REs, indicating that Rab35 is crucial for regulating the localization of MICAL-L1, which in turn acts as a scaffold for the Rabs in endosome recycling. Finally, Rab35 regulates the formation of an association site between the molecular scissor EHD1 and Arf6-positive endosomes by integrating the functions of two different Rab35 effectors for the successful growth of neurites (Kobayashi and Fukuda, 2013).

In PC12 cells, TBC1D12 (Rab11-binding protein) also modulates the growth of neurites (Oguchi et al., 2017) and is regulated by Rabin8 through coordination with Rab8, Rab10, and Rab11 and by an independent mechanism from GEF activity (Homma and Fukuda, 2016). With this in mind, Furusawa et al. proposed an interesting mechanism for regulating membrane transport in growing axons: GRAB (a Rab8-GEF and also a regulator of axon extension) promotes axonal membrane transport by mediating the interaction between Rab11 and Rab8 in neurons. In addition, GRAB activity is regulated by phosphorylation of Cdk5-p35, thus modulating axonal growth through the Rab11-GRAB-Rab8 cascade (Furusawa et al., 2017). Moreover, by using light-induced heterodimerization, it was proposed that growth cone dynamics and axon growth of rat hippocampal neurons directly depend on the functioning of the Rab11 vesicle near the growth cone, rather than the general functions of Rab11 in other parts of the cell (van Bergeijk et al., 2015). In dorsal root ganglion neurons, the expression of Rab11 increases neurite length, and the knockdown of Rab11 by siRNA decreases neurite outgrowth (Eva et al., 2010).

Finally, there are numerous reports implicating Arf6 as a central regulator for local actin polymerization and/or dynamics. As an example, the activation of Arf6 induces the recycling of Rac1 (Zobel et al., 2018) and controls actin polymerization mediated by a direct interaction with RhoB (Zaoui et al., 2019), and EFA6 protein (Arf6 GEF) can interact directly with F-actin promoting its polymerization (Macia et al., 2019). Although these mechanisms have not been described as regulating neuronal functions, it seems plausible that coordination between the membrane and the actin dynamics observed in other cell types may be essential to coordinate the local release of the membrane and the modifications of the cytoskeleton that support axonal elongation.

### Dendritic Growth

Initially, Arf6 and the Arf nucleotide-binding site opener (ARNO, which acts as Arf-GEF) were identified in the embryonic and adult hippocampus, as negative regulators of both the onset and branching of dendritic tree development, at 1–2 days *in vitro* (DIV) (Hernandez-Deviez et al., 2002). Later, the same authors expanded the described effects of these molecules to include the regulation of axonal elongation and branching during neuronal development, in early developmental stages (1–6 DIV) (Hernandez-Deviez et al., 2004). Subsequently, it was shown that signaling through ARNO is also necessary for Schwann cell myelination (Torii et al., 2015). Furthermore, Arf6-specific GAP (ACAP3) was shown to positively regulate neurite (axon and dendrites) growth in mouse hippocampal neurons (Miura et al., 2016).

Moreover, Rab11 has been reported to participate in the initiation, maintenance, and regulation of axonal and dendritic growth and synaptic transmission (Sann et al., 2009; Villarroel-Campos et al., 2016a). Results obtained in cortical neurons using constitutively active Rab11a-Q70L, but not dominant-negative Rab11a-S25N, showed the promotion of axonal growth (Takano et al., 2012). Subsequently, Takano et al. proposed that LMTK1 (lemur kinase 1A) negatively controlled dendrite growth and arborization, thus enhancing the movement of the Rab11a-positive endosome (to similar levels to those expressing Rab11A-Q70L) in a Cdk5-dependent manner (Takano et al., 2014). The dynamics of REs are regulated by BDNF (brain-derived neurotrophic factor) that increases Rab11 activity and recruits Rab11-positive vesicles for dendrites. Consistently, the overexpression of Rab11 in this context produces an increase in dendritic branching in neurons to 7 DIV (Lazo et al., 2013). On the contrary, neurons at 3 and 7 DIV show more complex dendritic arborization after Rab11 suppression, with an increase in the number of branching points and in the number of primary processes (arising directly from the soma) only at 3 DIV (Siri et al., 2020). The apparent discrepancy of phenotypes in the dendritic tree caused by Rab11 activity needs to be addressed by analyzing other stages of differentiation. Regarding Rab35, its participation in this process has not yet been described.

Since endo- and exocytosis mechanisms control essential features of receptors recycling controlling synaptic strength (Lamprecht and LeDoux, 2004), Arf6 functions linked to clathrin-dependent and independent endocytosis may provide light to uncover novel Arf6 functions coordinating dendritic remodeling and synaptic plasticity (Krauss et al., 2003).

### Migration of Cortical Neurons

*In situ* experiments using *in utero* electroporation show how Arf6 regulates neuronal migration in the developing cerebral cortex and highlights the physiological relevance of the Arf6-dependent membrane trafficking pathway in cortex development. Low levels of Arf6-GTP are necessary for the early stages of corticogenesis (Arvanitis et al., 2013), as increasing levels of active Arf6 cause delays in radial migration and defective terminal branches of projection neurons (Falace et al., 2014). In addition, the physiological importance of ACAP3 (Arf6-GAP) in brain development *in vivo* has been shown. The knockdown of ACAP3 in developing cortical neurons of mice significantly abrogates neuronal migration in the cortical layer, which is restored by the ectopic expression of ACAP3, but not by its inactive GAP mutant (Miura and Kanaho, 2017).

Moreover, Rab11-dependent recycling to promote neuronal migration along radial glial fibers is essential in enabling active N-cadherin transport in locomotor neurons in the cerebral cortex (Kawauchi et al., 2010). In this regard, it is important to note that FIP3 (family interaction protein Rab11 3 (FIP3)/Arfophilin-1, a dual effector for Arf6 and Rab11) is a regulator of N-cadherin traffic through interaction with Arf6 and Rab11 in migratory neurons (Hara et al., 2016).

Finally, it is unknown whether Rab35 is involved in neuronal migration, and further studies are required to explore this point.

### Synaptic Plasticity

Emerging evidence using *in vitro* and *in vivo* studies in hippocampal neurons has been shown that Arf6 (or Arf6-GEF or -GAP) regulates AMPA receptor trafficking and long-term synaptic plasticity at postsynaptic sites (Scholz et al., 2010; Myers et al., 2012; Oku and Huganir, 2013; Zheng et al., 2015). Moreover, an interesting report showed a new role for Arf6 in determining the size of releasable SVs and in promoting direct vs. endosomal recycling of these vesicles (Tagliatti et al., 2016). The location of Arf6 in the dendritic spines of mature neurons indicates that Arf6 is linked with synaptic plasticity. However, there are contradictory results in this regard, as Arf6-Q67L overexpression decreases the density of the spines, while the opposite result is obtained with the Arf6-T27N mutant in 21 DIV neurons (Miyazaki et al., 2005). Conversely, in 11 DIV neurons, activation of Arf6 (by overexpression of a fast-cycling Arf6 mutant—Arf6-T157A) increases spine density, whereas an Arf6 knockdown decreases spine formation (Choi et al., 2006). The answer to this controversy which was proposed by Kim et al. who suggested that the different abilities of Arf6 to regulate the formation and maintenance of the spine were related to maturation and neuronal activity: Arf6 activation increases the formation of the spine in developing neurons, yet it decreases the density of the spine in mature neurons (Kim et al., 2015).

Using high-resolution live-cell imaging, it was demonstrated that removal of Rab11 REs from dendritic spines decreases the level of AMPA receptors in the spine membrane and PSD-95 clusters in synapses, suggesting a mechanistic link between endosome positioning and the structure and composition of synapses (Esteves da Silva et al., 2015). Later, by investigating the putative regulators of endosomal trafficking involved in spinogenesis, many other molecules were identified. In this regard, TBC1D9B and LMTK1 (which controls the GAP activity of TBC1D9B in Rab11) have been proposed as novel factors that control spine formation by the Cdk5-LMTK1-TBC1D9B-Rab11 cascade (Nishino et al., 2019).

### Glial Differentiation and Myelination

Many aspects of glial cell differentiation are regulated by functions associated with trafficking. In fact, Rab35 and ACAP2 (Rab35-GAP that also inactivates Arf6 activity) have been shown to downregulate the morphological differentiation of oligodendrocytes (OL). Suppression of Rab35 or ACAP2 promotes OL differentiation. The knockdown of Arf6 inhibits differentiation, indicating that Rab35 and ACAP2 regulate differentiation by downregulation of Arf6. Furthermore, using neuronal OL cultures, the loss of Rab35 or ACAP2 was found to promote myelination, while the deletion of Arf6 inhibits myelination (Miyamoto et al., 2014). Interestingly, and because the complete loss of Arf6 results in embryonic lethality (Suzuki et al., 2006), a conditional knockout mouse (CKO) was generated which lacked Arf6 in neurons, OLs, or both cell lineages. Under these conditions, and consistent with the results mentioned above, axonal myelination during neuronal development *in vivo* was affected in the hippocampus fimbria and corpus callosum, but only in neuron-specific Arf6-CKO mice; Arf6 also regulates the migration of oligodendrocyte precursor cells (OPCs) (Akiyama et al., 2014). Furthermore, the lack of Arf6 specifically in mouse Schwann cells reduces myelin thickness, consistent with the negative regulation of signaling molecules related to myelination, demonstrating that Arf6 plays a key role in the myelination process (Torii et al., 2015).

Regarding Rab11, a positive regulation of this Rab and the importance of the SH3TC2 (effector of Rab11)/Rab11 interaction for normal myelination has been demonstrated in dorsal root ganglia (Stendel et al., 2010).

Finally, Rab35 in addition to recycling functions also participate in endocytic trafficking functionally connected to Arf6 (Kanno et al., 2010; Dambournet et al., 2011; Donaldson et al., 2016). Rab35 and Arf6 antagonism previously described seems to be essential to balance the activity of these two small GTPases to fine tune endocytosis.

**FIGURE 1:**
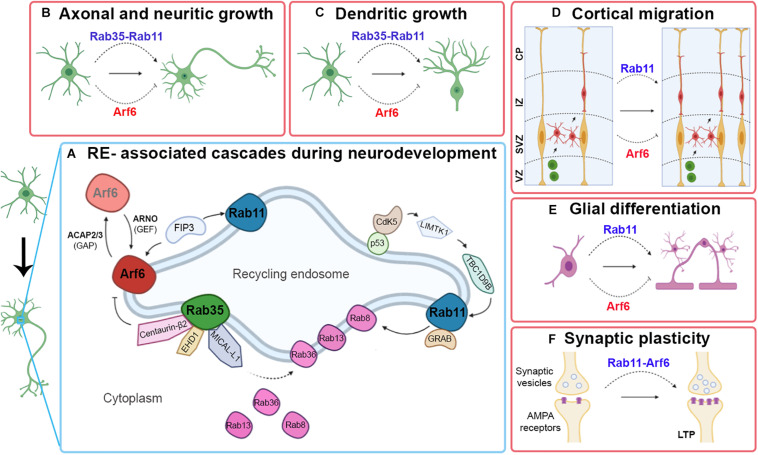
Contributions of Rab11, Rab35, and Arf6 RE-associated in certain events related to neuronal development. A simplified outline of some RE-associated signaling cascades to Rab11, Rab35, and Arf6 involved in neuronal development **(A)**. Schematic image showing the type of participation of Rab11, Rab35, and Arf6 RE-associated with axonal and neuritic growth **(B)**, dendritic growth **(C)**, cortical migration **(D)**, and glial differentiation **(E)**. Pointed arrows indicate positive regulation; blunt arrows indicate negative regulation.

In conclusion, throughout the different aspects analyzed during the neurodevelopment of mammals, it is possible to determine the active role played by both Rab35 and Rab11, always acting as positive regulators. In contrast, Arf6 is a negative regulator (Figure 1). It would be interesting to evaluate the signals that activate the negative regulation of Arf6 and if the similar roles of Rab35 and Rab11 occur by synergy or modulating different aspects.

## Rab11, Rab35, and Arf6 Re-Associated Functions During *Xenopus laevis* Neurodevelopment

TBC1d24, a Rab35 GAP, complexes with ephrinB2 via the scaffold Disheveled (Dsh) and mediates a signal affecting contact inhibition of locomotion in the cranial neural crest (CNC). Moreover, in the migrating CNC, the interaction between ephrinB2 and TBC1d24 negatively regulates E-cadherin recycling via Rab35 (Yoon et al., 2018).

Regarding Arf6, in *Xenopus* neurons at the neuromuscular junction, Arf6 modulates neurotransmitter release in a GEF msec 7-1-dependent fashion (Ashery et al., 1999).

Finally, Rab11 knockdown in rods leads to shortened outer segments and retinal degeneration, and the direct interaction between rhodopsin and Rab11 is required for the formation and maintenance of vertebrate photoreceptors (Reish et al., 2014).

## Rab11, Rab35, and Arf6 Re-Associated Functions During Zebrafish (*Danio Rerio*) Neurodevelopment

The modulation of Arf6 activity rescues interrupted traffic pathways at the start of photoreceptor development (George et al., 2016). Furthermore, zRab11-FIP4 (an ortholog of the Rab11-4 family interaction protein, Rab11-FIP4) is expressed predominantly in neural tissues, including the retina, and zRab11-FIP4 is involved in the regulation of proliferation and differentiation of retinal cells during development (Muto et al., 2006). Additional Rab11 genes (*rab11a*, *rab11ba*, and *rab11bb*) play vital and differential roles during Zebrafish embryonic development of the nervous system (Zhang et al., 2019).

## Rab11, Rab35, and Arf6 Re-Associated Functions During *Drosophila Melanogaster* Neurodevelopment

The participation of Rab35 in the formation of *Drosophila* led to the identification of Fascin, a protein that groups actin as an effector of Rab35 (Zhang et al., 2009; Shim et al., 2010). In this sense, the effect of nuclear Rab11 Fallout (Nuf) on actin remodeling during cytokinesis has also been demonstrated, probably by compromising Rho-GEF2-Rho1 (Cao et al., 2008). Although Rab35 and Rab11 modulate the actin cytoskeleton in different processes of *Drosophila* development, their possible complementary roles have not yet been explored.

Rab35 plays a critical role in the regulation of PtdIns (4,5) levels in phase 2 (P2) of cytokinesis in *Drosophila* (Kouranti et al., 2006). Inactivation of Rab35 using a Rab35 S22N dominant-negative mutant induces the formation of abnormal intracellular vacuoles rich in PtdIns (4,5) in P2. This defect leads to delocalization of SEPTIN2 a protein that binds to PtdIns (4,5) and subsequent accumulation of F-actin. The relocation of SEPTIN2 from the plasma membrane in these vacuoles possibly explains the instability of the excision groove and cytokinesis failure, two phenotypes observed after inactivation of Rab35. Rab35 is also downregulated by Arf6 during cytokinesis (Chesneau et al., 2012).

The knockdown of Schizo (Arf6-GEF like mammalian Arf-GEP100) produces misguidance of commissural axons, thus demonstrating the importance of Arf6 in axonal guidance *in vivo* (Onel et al., 2004). Recently, it was reported that in Arf6-deficient mice many commissural axons were stalled at the midline, reinforcing the initial observations in *Drosophila* (Kinoshita-Kawada et al., 2019).

Moreover, Rab11 is required for membrane trafficking and actomyosin ring constriction in meiotic cytokinesis of *Drosophila* males (Giansanti et al., 2007). Furthermore, during the differentiation of photoreceptor terminals, adequate traffic and the location of rhodopsin is crucial for the morphogenesis of the rhabdomere, and Rab11 activity has a key role in the initial delivery of exocytic rhodopsin to the growing rhabdomere (Satoh et al., 2005), The Parcas protein is the predominant Rab11-GEF required for rhodopsin transport (Otsuka et al., 2019). The translocation of photoreceptor (R cell) nuclei during *Drosophila* development is a model system used to analyze mechanisms controlling neuronal migration and positioning during embryonic development. The Rab5-Shibire/dynamin-Rab11-dependent vesicular transport pathway is involved in R-cell positioning (Houalla et al., 2010). The movement of R-cell nuclei along the apical–basal axis in the developing fly visual system displays features very similar to the somal translocation of neurons from the ventricular zone to the cortical plate during the development of the mammalian cerebral cortex (Nadarajah and Parnavelas, 2002).

Mutant embryos expressing dominant-negative or constitutively active Rab11 or carrying null Rab11 show disorganization and misdirected embryonic axons (Bhuin and Roy, 2009). In addition, Rab11 is required for pruning the c4da neuronal dendrites (sensory dendritic arborization class IV), since the loss of Rab11 produces defects in the development of the larval dendrite and also in the location of two neuronal membrane proteins, Nrg and Ppk26 (cell adhesion molecule and mechanosensory ion channel, respectively) (Kramer et al., 2019).

## Rab11, Rab35, and Arf6 Re-Associated Functions During *Caenorhabditis elegans* Neurodevelopment

In response to stress, UNC-70/β-spectrin, and TBC-10, a conserved GAP, stabilize the hemidesmosome’s trans-epidermal junction structures that would otherwise be lost, causing axonal rupture and degeneration. Furthermore, TBC-10 regulates axonal fixation and maintenance by inactivating RAB-35 and reveals the functional conservation of these molecules with vertebrate orthologs (Coakley et al., 2020).

Recent studies show RAB-11-interacting protein (REI-1) as a new GEF for RAB-11. The loss of REI-1 impairs targeting of RAB-11 to the late Golgi compartment, as well as recycling of endosomes in embryos, and further reduces RAB-11 recruitment in the excision sulcus, retarding cytokinesis (Sakaguchi et al., 2015).

## Concluding Remarks

The summarized evidence reveals the role of recycling endosomes in different developmental steps and animal models and that clearly one Rab does not rule them all. The RE data from Rab11, Rab35, and Arf6 suggest that heterogeneous domains of REs work synergistically, in some cases, and with opposite roles in others (Figure 1). Regarding this point, it would be interesting to understand the signals and the environmental requirements that allow it, as well as the possibility of a redundant function of these REs.

Furthermore, the presence and participation of these REs in several animal models throughout evolution are clear, indicating the relevance of these endosomes in functions that are conserved from lower to greater complexity levels in the nervous system.

Finally, the importance and the complex participation of other RE-resident Rab proteins required for neuronal development and synaptic function have been demonstrated in several publications and we summarized in Table 1. However, some questions remain unclear: how do these Rab orchestrate the different developmental processes? How are the signaling cascades linked to regulating each other? Understanding the coordination that these Rabs carry out for the correct establishment of sophisticated neuronal morphology and specialized compartmentalization is crucial for a better understanding of various aspects of neuronal physiology and pathophysiology.

**TABLE 1:** Main contributions of Rab RE-associated with neuronal development.

RE-resident	Endosomal			
Rab/Ras	localization	Main contribution to neuronal development	Experimental model	References
Rab4	EE, RE	Promotes axon elongation.	*Xenopus laevis*	Falk et al., 2014
		Critical for maintaining dendritic spine size.	Rat hippocampal neurons	Brown et al., 2007*;* Hoogenraad et al., 2010
		Regulates synapse homeostasis through kinesin-2 mediated trafficking.	*Drosophila*	Dey et al., 2017
Rab8	TGN, RE	Involved in the transport of proteins to the dendritic surface and in neurite outgrowth.	Rat hippocampal neurons	Huber et al., 1993, 1995
		Required for the local delivery of AMPARs into synapses.	Rat hippocampal neurons	Gerges et al., 2004
		Regulates axonal outgrowth via GRAB-mediated Rab8-Rab11 cascade in a Cdk5-dependent manner.	Mouse cortical neurons	Furusawa et al., 2017
Rab11	RE	Promotes neurite/axonal elongation and axon regeneration.	Rat cortical neurons	Takano et al., 2012*;* Koseki et al., 2017;
		Promotes AMPARs and PSD-95 clusters at the synapses along actin and microtubule cytoskeleton.	Rat hippocampal neurons	Esteves da Silva et al., 2015
		Involved in dendritic branching and spatial memory formation.	Rat hippocampal neurons	Siri et al., 2020
		Participates in N-Cadherin trafficking regulating neuronal migration and maturation.	Mouse cerebral cortex	Kawauchi et al., 2010
		Required for actin cytoskeleton remodeling during early *Drosophila* furrow formation.	*Drosophila*	Riggs et al., 2003
		Required for the development of the outer segment of rod cell membranes.	Mouse retina cells	Reish et al., 2014
		Involved in Zebrafish embryonic differentiation and development of the nervous system.	Zebrafish	Zhang et al., 2019
Rab13	TGN, RE	Increases neurite outgrowth.	PC12; Mouse dorsal root ganglion neurons	Di Giovanni et al., 2005; Kobayashi et al., 2014
		Involved in the reorganization of the actin cytoskeleton through the Rab13-JRAB/MICAL-L2 interaction.	PC12	Sakane et al., 2010
Rab17	EE, RE	Regulates dendrite morphogenesis and postsynaptic development.	Mouse hippocampal neurons	Mori et al., 2012, 2013
		Regulates dendritic surface insertion of GluK2-containing KARs by dendritic trafficking of Syntaxin-4.	Rat hippocampal neurons; Neuro2A	Mori et al., 2014
Rab35	RE	Promotes neurite outgrowth.	PC12; N1E-115	Chevallier et al., 2009*;* Kobayashi and Fukuda, 2013
		Regulates membrane trafficking from recycling endosomes to neurite tips during neurite outgrowth.	PC12	Kobayashi et al., 2014
		Regulates axonal elongation and Cdc42 activity in neurons.	Rat hippocampal neurons	Villarroel-Campos et al., 2016b
		Implicated on sorting of synaptic vesicle proteins in neuromuscular junctions.	*Drosophila*	Uytterhoeven et al., 2011
		Involved in maintain axonal integrity via UNC-70/β-spectrin-TBC10-Rab35.	*Caenorhabditis elegans*	Coakley et al., 2020
Arf6	RE	Inhibits neurite/dendritic/axonal elongation and branching during neuronal development.	PC12; Chick retinal and rat hippocampal neurons; Aplysia motor neurons	Hernandez-Deviez et al., 2002, 2004; Albertinazzi et al., 2003; Huh et al., 2003; Kobayashi and Fukuda, 2012
		Enhances clathrin/AP-2 recruitment at the synapse by PIPKIγ activation.	Rat cortical neurons	Krauss et al., 2003
		Regulates the formation and maintenance of the dendritic spines.	Rat hippocampal neurons	Miyazaki et al., 2005; Choi et al., 2006; Kim et al., 2015
		Regulates neuronal migration in the developing cerebral cortex.	Mouse cerebral cortex	Arvanitis et al., 2013; Falace et al., 2014
		Promote neurotransmitter release at the neuromuscular junction.	Xenopus laevis	Ashery et al., 1999
		Regulates traffic pathways during photoreceptor development.	Zebrafish	George et al., 2016

*The table summarizes the role of RE-resident Rabs/Ras reported in processes mainly associated with neuronal differentiation and migration of several experimental models.*

## Author Contributions

VR-S, CG-B, and CC wrote and edited the manuscript. All authors contributed to the article and approved the submitted version.

## Conflict of Interest

The authors declare that the research was conducted in the absence of any commercial or financial relationships that could be construed as a potential conflict of interest.
